# Lupus Enteritis as the Early Manifestation of Systemic Lupus Erythematosus Successfully Managed With Belimumab: A Case Report

**DOI:** 10.7759/cureus.76926

**Published:** 2025-01-04

**Authors:** Rawan Almutairi, Dalal Alkhudair, Ali Aldei

**Affiliations:** 1 Dermatology, Farwaniya Hospital, Ministry of Health, Farwaniya, KWT; 2 Rheumatology, Amiri Hospital, Kuwait City, KWT

**Keywords:** gastroenteritis, lupus enteritis, sle initial diagnosis, systemic lupus erythematosis, systemic lupus erythematosus

## Abstract

Lupus enteritis (LE) is an uncommon cause of abdominal pain in individuals with systemic lupus erythematosus (SLE). This condition is explained by the inflammation of the intestinal wall caused by the accumulation of immune complexes and activation of the complement system. A 19-year-old previously healthy Kuwaiti woman arrived at the emergency department (ED) complaining of diffuse abdominal pain, non-bloody diarrhea, and nausea. The symptoms started two days before presentation. The patient described the abdominal pain as sudden, sharp, and stabbing in nature, with a severity of 10 out of 10, and mainly located in the suprapubic area. On examination, the patient’s vital signs were normal. She had no skin rash, oral ulcers, arthritis, or palpable lymphadenopathy. Her abdomen was soft on palpation, with diffuse tenderness. Her initial laboratory investigations showed a normal hemoglobin level and WBC count but lymphopenia (0.6 x 10^9^/L; normal, 1-3 x 10^9^/L). A computed tomography (CT) scan of the abdomen showed small bowel loops with diffuse edematous wall thickening with target signs. The descending and sigmoid colon, and the rectum walls were also thickened and edematous. Significant pericolonic fat stranding and free fluid with preserved enhancement suggested an active systemic inflammatory process. Anti-nuclear antibody and anti-double stranded DNA antibody test results were positive. Acute abdominal pain was managed with IV corticosteroids, and 10 mg/kg IV belimumab was initiated. The patient was followed up in OPD after one, three, and six months, where she did not mention any relapse of LE nor any side effects from belimumab. The diagnosis of LE in this case was challenging because of the absence of a prior diagnosis of SLE. Clinical manifestations of this condition, which include abdominal discomfort, vomiting, and diarrhea, are non-specific and can be mistaken for several chronic gastrointestinal disorders, posing a diagnostic problem. Although belimumab is not commonly used to treat LE, we were able to successfully manage the patient using it, making it a promising new method for treating LE.

## Introduction

Lupus enteritis (LE) is an uncommon cause of abdominal pain in individuals with systemic lupus erythematosus (SLE) [1]. It has been described using several terms including lupus mesenteric vasculitis, gastrointestinal vasculitis, intra-abdominal vasculitis, and acute gastrointestinal syndrome. This condition is characterized by inflammation of the intestinal wall caused by the accumulation of immune complexes and activation of the complement system. This leads to vasculitis and damage owing to reduced blood flow [2]. The worldwide prevalence of LE varies from 0.2% to 9.7% among all patients with SLE, and from 29% to 65% among SLE patients with acute abdominal discomfort [3]. The suggested causative factors include bacterial infections that modify the composition of intestinal flora, cytomegalovirus infection, eosinophilia, non-steroidal anti-inflammatory drugs, chemicals, metallic particulates, animal viruses, helminth infection, caffeine, phosphodiesterase-4 inhibitors, adenosine diphosphate, specific foods, and herbal medicines [4]. Investigating LE is crucial because of its potential to be misdiagnosed as illnesses that have high mortality and morbidity rates, such as mesenteric ischemia, intestinal obstruction, perforation, and other prevalent causes of abdominal pain. Here, we report a rare case of LE as the first manifestation of SLE that was well managed with belimumab.

## Case presentation

A 19-year-old previously healthy Kuwaiti woman presented to the emergency department (ED) complaining of diffuse abdominal pain, non-bloody diarrhea, and nausea, with the symptoms starting two days before presentation. The patient described the abdominal pain, located mainly in the suprapubic area, as sudden, sharp, and stabbing in nature, with a severity of 10 out of 10. She denied any history of joint pain, rash, photosensitivity, alopecia, sicca symptoms, oral ulcers, genital ulcers, Raynaud’s phenomenon, red color urine, pleuritic chest pain, shortness of breath, or fever; she was not on menses. The remainder of her systemic review was normal, and she was not taking any regular medication.

In the last three months, she had visited ED with a similar presentation of enteritis symptoms including abdominal pain and vomiting, which were managed successfully with supportive intravenous fluids, anti-emetics, and antibiotics. Her family history revealed no rheumatological disease in the family, such as lupus erythematosus or rheumatoid arthritis. She was a non-smoker and non-alcoholic.

On presentation, the patient’s vital signs were normal. She had no skin rash, oral ulcers, arthritis, or palpable lymphadenopathy. The results of the neurological, cardiovascular, and pulmonary examinations were normal. The abdomen was soft on palpation, with diffuse tenderness, but more in the suprapubic area without guarding. Table 1 shows her laboratory investigation results.

**Table 1:** Laboratory investigations Liver, renal, and thyroid function tests, and coagulation profile were all normal. Urinalysis and plasma lactic acid level were also normal.

	Value	Normal range
White blood cells	9	4-11 x 10^9^/L
Lymphocytes	0.6	1-3 x 10^9^/L
Hemoglobin	12.5	11.5-15.5 g/L
Platelets	189	150-450 x 10^9^/L
Stool Clostridioides difficile toxin	Negative	
Stool routine and culture	Negative	
C-reactive protein	21.7	0.3-1.0 mg/dL
Anti-nuclear antibody	1:640	1:40
Anti-double-stranded DNA antibodies	666.9	0-35 IU/mL
Anti-SSA/Ro	Positive	
Anti-SSB/La	Positive	
C3	0.76 g/L	0.8-1.7 g/L
C4	0.07 g/L	0.15-0.45 g/L
IgG	36	8-18 g/L

**Figure 1:**
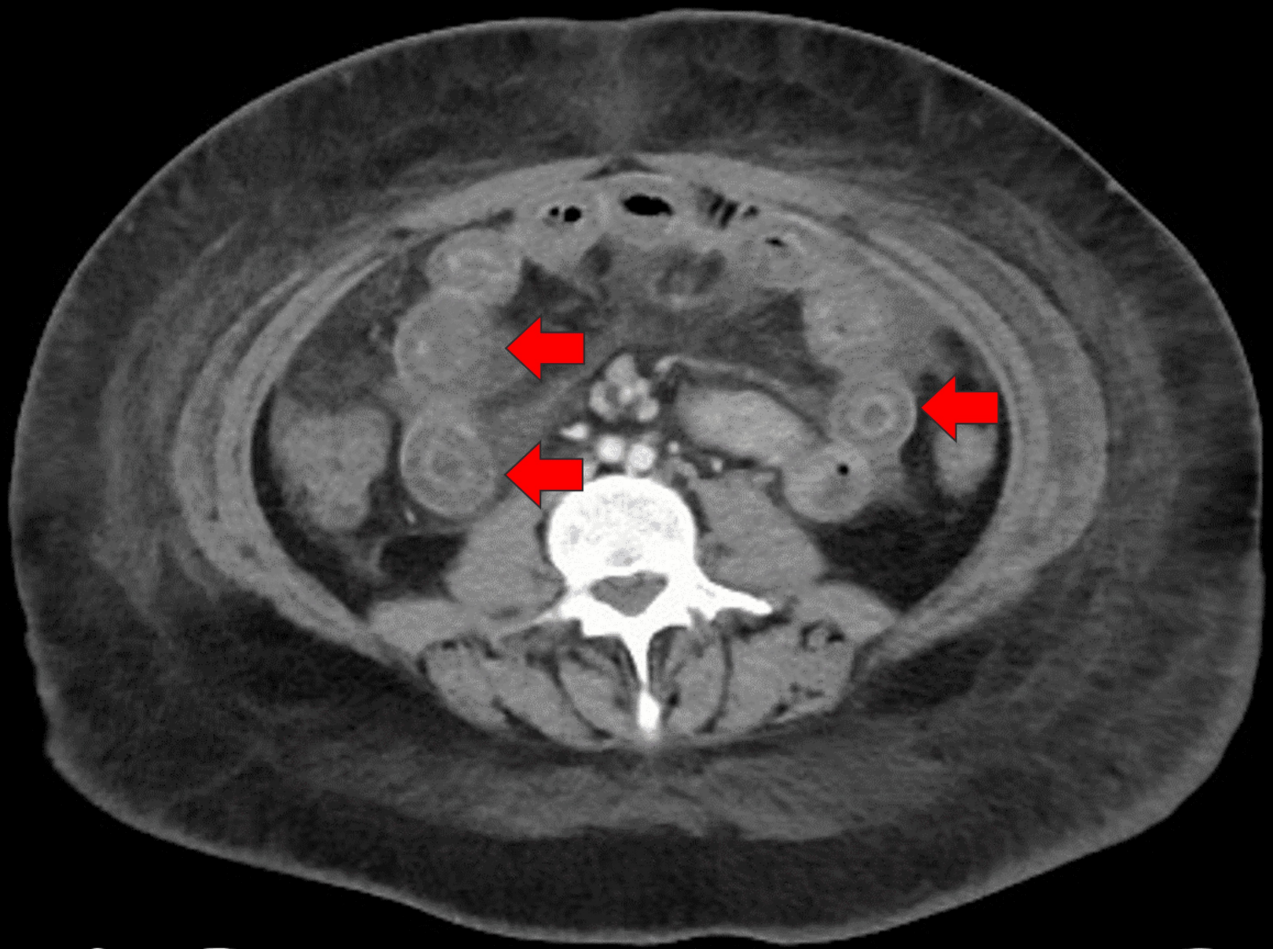
Abdominal CT scan (axial view) Small bowel loops showed diffuse edematous wall thickening with target signs (red arrows).

**Figure 2:**
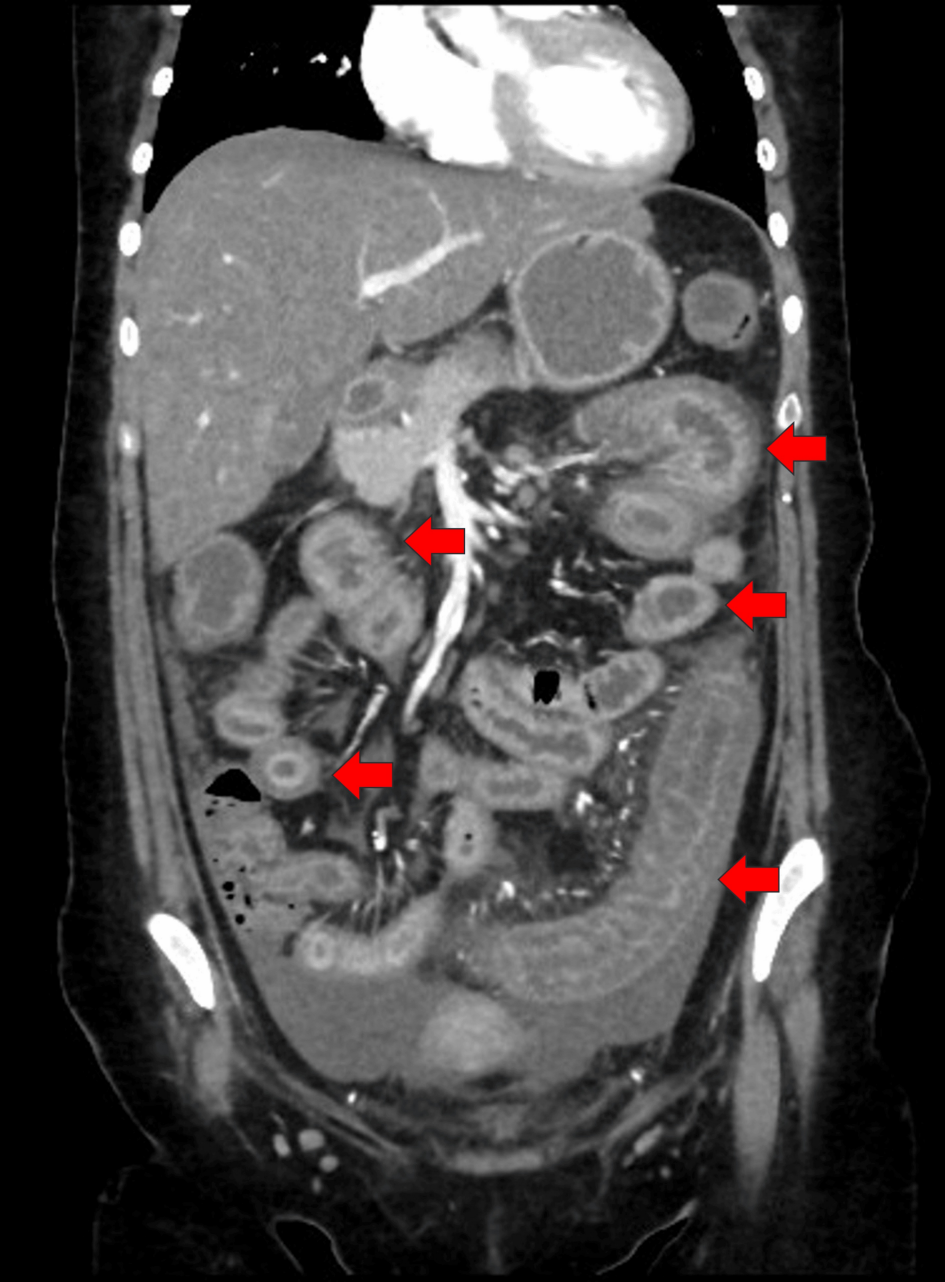
Abdominal CT scan (coronal view) Descending and sigmoid colon, and the rectum walls were thickened and edematous (red arrows); significant pericolonic fat stranding and free fluid with preserved enhancement suggested an active systemic inflammatory process.

Despite the symptomatic treatment with antibiotics and proton pump inhibitors, the patient did not show any improvement, which necessitated hospital admission for further investigations and proper management. A CT scan of the abdomen revealed small bowel loops that showed diffuse edematous wall thickening with target signs (Figure 1). The descending and sigmoid colon, and the rectum walls were thickened and edematous. Significant pericolonic fat stranding and free fluid with preserved enhancement were suggestive of an active systemic inflammatory process (Figure 2). Colonoscopy and upper endoscopy were done and revealed mild gastritis with otherwise normal findings; a biopsy showed chronic gastritis and duodenitis with Brunner’s gland hyperplasia, without evidence of *Helicobacter pylori* or intestinal metaplasia.

The extractable nuclear antigen test was positive for lupus erythematous antibodies, namely, anti-nuclear antibody (ANA) and anti-double-stranded DNA antibodies with high titers at 1:640 and >666.9 (reference range, 0-35 IU/mL), respectively. In addition, the patient tested positive for anti-SSA/Ro and anti-SSB/La, and had low complement levels, with C3 at 0.76 g/L and C4 at 0.07 g/L. Moreover, the patient had an elevated level of IgG at 36 g/L (reference range, 8-18 g/L); other immunoglobins were normal.

Considering the patient's history, and laboratory and radiological findings, she was diagnosed with LE and was started on IV methylprednisolone 1 mg/kg for five days with significant improvement in her gastrointestinal symptoms. She was then shifted to hydroxychloroquine 200 mg daily, azathioprine 50 mg daily, and prednisone 60 mg daily, tapered off over two months. The patient showed no abdominal symptoms during the two-month follow-up. Yet, she mentioned having SLE manifestations, photosensitivity and arthritis.

After two more months, despite being compliant with her medications, she presented to the ED with the same enteritis symptoms, including severe abdominal pain, diarrhea, and vomiting. A CT scan showed the same findings that suggested LE. Symptoms were managed successfully with IV methylprednisolone 1 mg/kg for five days. After one week, she was much better with minimum abdominal pain. Azathioprine 50 mg was stopped and IV belimumab 10 mg/kg was initiated and given every two weeks for the first three doses and then every month for the next doses. The patient was followed up in the OPD after one, three, and six months, where she did not mention any relapse of LE nor any side effects from belimumab.

## Discussion

LE is characterized by a range of non-specific clinical manifestations, including abdominal pain (97%), ascites (78%), nausea (49%), vomiting (42%), diarrhea (32%), and fever (20%) [5]. Moreover, SLE patients with gastrointestinal manifestations commonly have a high SLE Disease Activity Index [6]; however, our patient denied symptoms of other SLE manifestations upon first presentation. SLE was not one of the differential diagnoses, which undoubtedly made the diagnosis challenging.

The most frequent laboratory findings detected in patients with LE include hematologic findings such as leukopenia, lymphopenia, and anemia. Additionally, these patients often test positive for ANA in 92% of cases, anti-double-stranded DNA antibodies in 74% of cases, low levels of complement proteins in 88% of cases, anti-ribonucleoprotein antibodies in 28% of cases, anti-SSA in 26% of cases, and anti-Smith antibodies in 24% of cases. Elevated CRP levels are not a typical feature of this condition [1,5,7].

It is crucial to exclude the possibility of concurrent lupus nephritis, which is found in 65% of all cases of LE and seems to coexist in most instances of SLE that initially presents with LE [8,9]. Our patient's lupus nephritis investigations were negative.

SLE is distinguished by the significant presence of autoantibodies, specifically IgG, which plays a role in the development of inflammation and damage to organs and tissues. Furthermore, the degree of tissue injury is associated with the amount of IgG deposited [10]. Our patient had a high level of IgG that could be related to the LE activity.

Brunner's gland hyperplasia is a benign growth in the duodenum that is seldom associated with apparent symptoms and accounts for 10.6% of benign tumors in the duodenum. It is believed to be caused by chronic inflammation, increased secretion of gastric acids, or the loss of pancreatic exocrine function [11]. Brunner’s gland hyperplasia of the duodenum was detected in our patient during endoscopy for the further assessment of abdominal pain. We believe that multiple attacks of LE resulted in hyperplasia of the Brunner’s gland.

Abdominal CT is necessary for the diagnosis of LE. The predominant imaging features that are observed include engorgement of mesenteric arteries (comb sign), heightened density of mesenteric fat, edema and thickening of the bowel wall, enlargement of intestinal segments, and the presence of a target sign [12], as identified in our patient.

Corticosteroids are the initial line of treatment for LE, and the illness frequently responds well to corticosteroids. In situations of severe disease, other organ involvement, or resistance, further immunosuppressive drugs, including cyclophosphamide, mycophenolate mofetil, or rituximab, may be administered [13]. Our patient did not show improvement with mycophenolate mofetil treatment. Nevertheless, the gastrointestinal symptoms were completely resolved, and there was no subsequent recurrence of gastrointestinal episodes after treatment with belimumab.

Belimumab is a monoclonal antibody approved for the treatment of non-renal lupus that targets B-cell survival factor (BAFF). The results of Phase III trials have demonstrated its efficacy in reducing overall disease activity and the occurrence and severity of flares without causing any deterioration in patients' overall state or the emergence of significant disease activity in new organ systems [14]. Belimumab has been reported to have a low frequency of side effects, good overall tolerability, and a favorable safety profile. Some of the most commonly reported side effects include arthralgia, upper respiratory tract infections, headaches, fatigue, and nausea [15].

## Conclusions

The diagnosis of LE in this case was challenging because of the absence of a prior diagnosis of SLE. The clinical manifestations of this condition, which include abdominal discomfort, vomiting, and diarrhea, are non-specific and can be mistaken for several chronic gastrointestinal disorders, posing a diagnostic problem. Moreover, in rheumatology, SLE can sometimes be easily identified because it may overlap with other immune disorders. But in our case, this aspect was absent making the diagnostic procedure more difficult. Belimumab is not commonly used to treat LE; however, we successfully managed the patient using the treatment option, making it a promising new method for treating LE.
